# The soluble (pro)renin receptor promotes a preeclampsia-like phenotype both in vitro and in vivo

**DOI:** 10.1038/s41440-024-01678-8

**Published:** 2024-04-11

**Authors:** Lachlan G. Schofield, Sarah J. Delforce, Jennifer C. Pryor, Saije K. Endacott, Eugenie R. Lumbers, Sarah A. Marshall, Kirsty G. Pringle

**Affiliations:** 1 School of Biomedical Sciences and Pharmacy, College of Health, Medicine and Wellbeing, University of Newcastle, Callaghan, NSW 2308 Australia; 2 Mothers and Babies Research Program, Hunter Medical Research Institute, New Lambton Heights, NSW 2305 Australia; 3 Immune Health Research Program, Hunter Medical Research Institute, New Lambton Heights, NSW Australia; 4 National Health & Medical Research Council (NHMRC) Centre of Research Excellence in Digestive Health, University of Newcastle, Newcastle, NSW Australia; 5 Department of Obstetrics and Gynaecology, The Ritchie Centre, School of Clinical Sciences, Monash University and The Hudson Institute of Medical Research, Clayton, VIC Australia

**Keywords:** Soluble (Pro)renin Receptor, Preeclampsia, Endothelial Dysfunction

## Abstract

Preeclampsia is classified as new-onset hypertension coupled with gross endothelial dysfunction. Placental (pro)renin receptor ((P)RR) and plasma soluble (P)RR (s(P)RR) are elevated in patients with preeclampsia. Thus, we aimed to interrogate the role (P)RR may play in the pathogenesis of preeclampsia. Human uterine microvascular endothelial cells (HUtMECs, *n* = 4) were cultured with either; vehicle (PBS), 25–100 nM recombinant s(P)RR, or 10 ng/ml TNF-a (positive control) for 24 h. Conditioned media and cells were assessed for endothelial dysfunction markers via qPCR, ELISA, and immunoblot. Angiogenic capacity was assessed through tube formation and adhesion assays. Additionally, pregnant rats were injected with an adenovirus overexpressing s(P)RR from mid-pregnancy (day 8.5), until term (*n* = 6–7 dams/treatment). Maternal and fetal tissues were assessed. HUtMECs treated with recombinant s(P)RR displayed increased expression of endothelial dysfunction makers including vascular cell adhesion molecule-1, intracellular adhesion molecule-1, and endothelin-1 mRNA expression (*P* = 0.003, *P* = 0.001, *P* = 0.009, respectively), along with elevated endothelin-1 protein secretion (*P* < 0.001) compared with controls. Recombinant s(P)RR impaired angiogenic capacity decreasing the number of branches, total branch length, and mesh area (*P* < 0.001, *P* = 0.004, and *P* = 0.009, respectively), while also increasing vascular adhesion (*P* = 0.032). +ADV rats exhibited increased systolic (*P* = 0.001), diastolic (*P* = 0.010), and mean arterial pressures (*P* = 0.012), compared with -ADV pregnancies. Renal arteries from +ADV-treated rats had decreased sensitivity to acetylcholine-induced relaxation (*P* = 0.030), compared with -ADV pregnancies. Our data show that treatment with s(P)RR caused hypertension and growth restriction in vivo and caused marked endothelial dysfunction in vitro. These findings demonstrate the significant adverse actions of s(P)RR on vascular dysfunction that is characteristic of the preeclamptic phenotype.

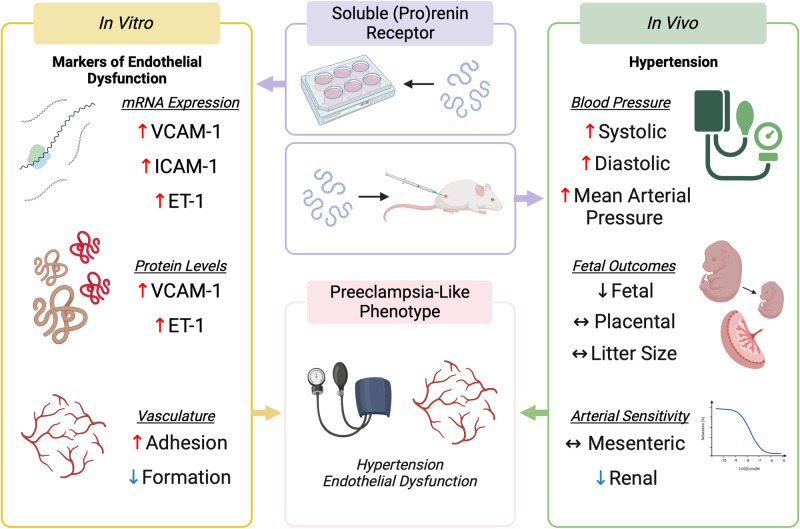

## Introduction

The soluble (pro)renin receptor (s(P)RR: *encoded by the ATP6AP2 gene*) is a component of the circulating renin-angiotensin system (RAS). Proteases (including furin [1, 2], ADAM19 [3], and site 1 protease (MBTPS1) [2, 4]) mediate the cleavage of the extracellular domain from full length (P)RR in the Golgi apparatus [1]. The newly released extracellular domain, known as the s(P)RR, is detectable in both plasma [1] and urine [5], where it can bind and activate prorenin and enhance its catalytic activity [6, 7].

Compared with normotensive pregnancies, aberrant (P)RR expression has been reported in preeclamptic pregnancies. Both full-length placental (P)RR and plasma s(P)RR levels are elevated in patients with preeclampsia, and they both correlate with systolic blood pressure in pregnant women [8]. Additionally, maternal plasma s(P)RR levels are elevated in pregnancies complicated by gestational diabetes mellitus [9, 10]. Previous studies by Morosin et al. have shown that primary human trophoblasts, isolated from term placentae and cultured in vitro, secrete s(P)RR [11]. Therefore, the high levels of s(P)RR found in the maternal circulation from women with preeclampsia, may be originating from the placenta.

Currently, the functional role of s(P)RR in pregnancy remains largely uncharacterised. Classically, the s(P)RR is considered to be involved in the RAS whereby the s(P)RR can bind to both renin, the rate-limiting enzyme of the RAS, and prorenin, the inactive precursor of renin [1, 6, 7]. As such, the s(P)RR can enhance the activity of the circulating and tissue-based RAS signalling pathways and more specifically, the intrarenal RAS [1]. Interestingly, the s(P)RR has also been demonstrated to be a direct agonist for the angiotensin II receptor type 1 (AT_1_R) [12], disrupting the notion of angiotensin peptides being the sole activating ligands for the AT_1_R [13, 14]. Increased stimulation of the AT_1_R contributes to the pathogenesis of cardiovascular disease, including hypertension [15]. Throughout normotensive pregnancy however the pressor response of angiotensin II is markedly blunted [16]. Recently, Fu et al. showed that direct binding of the s(P)RR with AT_1_R leads to the suppression of nitric oxide (NO) production in endothelial cells [12]. Throughout normotensive pregnancy, NO plays an important anti-inflammatory role as well as its roles in vasodilation and vasculogenesis/angiogenesis [17, 18]. Additionally, maternal serum leptin concentrations are higher in preeclampsia compared to normotensive pregnancies [19]. Recently, Gatineau et al. highlighted that high fat-fed male mice with elevated circulating s(P)RR had increased plasma leptin levels [20], potentially impairing baroreflex sensitivity and increasing systolic blood pressure. Taken together these data suggest that the elevated s(P)RR in preeclampsia could cause vascular endothelial dysfunction and hypertension [12, 17].

Due to the known and postulated effects of s(P)RR, we hypothesise that it may play a role in the regulation of blood pressure and endothelial function throughout pregnancy. In this study we have carried out experiments showing that s(P)RR elevates the blood pressure of pregnant rats and causes endothelial dysfunction in vitro, which supports the hypothesis that s(P)RR plays a role in the pathogenesis of preeclampsia.

## Methods

### Ethics approvals

#### Cell line

Ethics approval was obtained from the University of Newcastle Human Research Ethics Committee (H-2020-0398) to carry out work in the HUtMEC cell line.

#### Animal model

All animal experiments were approved by the Monash Medical Centre Animal Ethics Committee (MMCB2017/37) and conducted in accordance with the Australian Code of Practice and the National Health and Medical Research Council. Rats (Sprague Dawley, aged 3–6 months) were maintained on a 12 h light:12 h dark cycle at 20 °C, with standard food pellets and water available *ad libitum*.

### Culture of HUtMEC cell line

HUtMECs (Human uterine microvascular endothelial cells), purchased from Promocell (C-12295), are cells isolated from the myometrium of a single human donor. The HUtMEC cell line was chosen for this study as it was thought to be more reflective of the maternal vasculature than other commonly used endothelial cell lines, such as human umbilical vein endothelial cells (HUVECs), which are derived from the umbilical vein. HUtMECs were cultured in EBM-2 media (Lonza, CC-3156) supplemented with 5% fetal bovine serum (FBS), 0.04% hydrocortisone, 0.4% human fibroblastic growth factor (hFGF-B), 0.1% vascular endothelial growth factor (VEGF), 0.1% insulin-like growth factor 1 (R3-IGF-1), 0.1% ascorbic acid, 0.1% human epidermal growth factor (hEGF), 0.1% GA-1000 (Lonza; CC-4147) in a humidified incubator at 5% CO_2_ at 37 °C.

### Isolation of peripheral blood mononuclear cells

Peripheral blood mononuclear cells (PBMCs) were isolated from heparinised whole blood through density gradient centrifugation. Leucosep tubes (Greiner Bio-One, Kremsmünster, Austria) were prefilled with 15 mL of Lymphoprep separation medium (STEMCELL Technologies, Vancouver, BC, Canada) and briefly centrifuged. Blood was then added to the Leucosep tube and centrifuged at 800 × G for 15 min at room temperature (RT). The enriched cell fraction was then collected and washed twice with phosphate-buffered saline (PBS).

### Purification and Isolation of s(P)RR-FLAG protein

Briefly, HEK293 cells underwent transfection to incorporate plasmid DNA encoding s(P)RR and cell culture medium was collected [21, 22]. The soluble (pro)renin receptor (s(P)RR-FLAG) was isolated using affinity chromatography (α-FLAG-IgG) followed by size exclusion chromatography under low endotoxin conditions. Purified sPRR-FLAG was quantified and characterised by nanodrop (A_280_), SDS-PAGE and western blot (data not shown). The product was 266 amino acids (29,480.44 Da) with the following amino acid sequence:

#### Signal peptide

MAVFVVLLALVAGVLG

#### Protein region of interest

NEFSILKSPGSVVFRNGNWPIPGERIPDVAALSMGFSVKEDLSWPGLAVGNLFHRPRATVMVMVKGVNKLALPPGSVISYPLENAVPFSLDSVANSIHSLFSEETPVVLQLAPSEERVYMVGKANSVFEDLSVTLRQLRNRLFQENSVLSSLPLNSLSRNNEVDLLFLSELQVLHDISSLLSRHKHLAKDHSPDLYSLELAGLDEIGKRYGEDSEQFRDASKILVDALQKFADDMYSLYGGNAVVELVTVKSFDTSLI.

#### Flag peptide

DYKDDDDK.

### Treatment of HUtMECs with s(P)RR

HUtMECs were seeded at 1.75 × 10^4^ cells/well in 6 well plates with 2 ml of complete media without antibiotics and cultured at 37 °C in room air. After seeding cells for 24 h, cells were treated with one of five treatment groups containing, vehicle (media-only control), 25, 50 or 100 nmol/L s(P)RR-Flag protein (CSIRO, Clayton Australia), or 10 ng/mL TNF-α (positive control; Gibco Cat: RTNFAI, Lot:2285310) [23–25]. HUtMECs were treated for 24 h before cells and supernatant were collected, snap frozen in liquid nitrogen, and stored at −80 °C (N = 3–4 independent experiments performed in technical triplicate).

### Quantitative real-time polymerase chain reaction (qPCR)

RNA from HUtMECs was extracted using TRIzol (Life Technologies, California USA), following the manufacturer’s instructions. RNA integrity was validated using agarose gel electrophoresis (data not shown). A nanodrop ND-100 spectrophotometer was utilised to assess total RNA quantity and purity using *A*_260_/*A*_280_ and *A*_260_/*A*_230_ ratios. Total RNA from HUtMECs was reverse transcribed using a Superscript IV Reverse Transcriptase kit with random hexamers (Invitrogen, Massachusetts USA).

Real-time qPCR was performed using an Applied Biosystems Quant Studio 6 Flex real-time PCR system, using SYBR green for detection. Each reaction contained 5 µL of SYBR Green PCR master mix (Life Technologies), primers (Supplementary Table 1), 10 ng cDNA and water to 10 µL. The mRNA abundance of target genes in the HUtMECs was compared to the relative geomean of *ACTB* (β-actin), *B2M* (β-2 macroglobulin), and *YWHAZ* (Tyrosine 3-Monooxygenase/Tryptophan 5-Monooxygenase Activation Protein Zeta). The expression of the housekeepers (*ACTB, B2M*, and *YWHAZ*) did not change between treatment groups. Relative abundance was determined using the 2^−^^ΔΔCT^ method.

### Protein extraction and immunoblotting

Protein from HUtMECs was extracted using TRIzol, following the manufacturer’s instructions, before protein quantity was determined using a Pierce BCA protein assay kit (ThermoFisher Scientific, Massachusetts USA).

Using 10 μg per well, protein extracts underwent electrophoresis on a 4–12% Bis-Tris protein gel (NuPAGE, ThermoFisher Scientific). Proteins were transferred onto polyvinylidene difluoride (PVDF) membranes using the XCell Sure-Lock Mini-Cell electrophoresis system. Membranes were probed for VCAM-1 as follows: each membrane was blocked for 2 h at RT using 5% skim milk powder in tris-buffered saline (TBS) with 0.1% tween-20 (TBST). Membranes were then incubated at overnight at 4 °C with a VCAM-1 primary antibody (2 μg/ml, at 1:100 5% skim in TBST, SC13160, Santa Cruz), followed by a 2 h incubation with a horseradish peroxidase (HRP) conjugated goat anti-mouse secondary antibody (0.04 μg/ml, at 1:5000 5% skim in TBST, CS-7076, Cell Signalling).

Membranes were then stripped in 0.2 M sodium hydroxide before being re-blocked for 1 h at RT in 5% skim milk in TBST. Membranes were then re-probed for β-actin (ACTB) (0.2 μg/ml, at 1:5000 5% skim TBST, ab8227, Abcam) followed by a 1 h incubation with a HRP conjugated anti-rabbit secondary antibody (0.2 μg/ml, at 1:5000 5% skim TBST, 12-348, Merck-Millipore). The density of each band (determined by the Amersham Imager 600 analysis software) was corrected for its respective loading control (ACTB). Samples were run in duplicate, and the average calculated for the final analysis.

### HUtMEC enzyme-linked immunosorbent assays (ELISAs)

Commercial ELISA kits were used to measure ET-1 (Invitrogen, EIAET1), IL-6 (RnD Systems, DY206-05), and TNF-α (R&D Systems, DY210-05) in conditioned HUtMEC culture media according to the manufacturer’s instructions (Supplementary Table 2). A SPECTROstar^Nano^ micro-plate reader was utilised to measure the optical density at 450 nm for each sample. Sample dilution buffer was used to correct for background intensity.

### HUtMEC angiogenesis assay

Prior to the seeding of cells, 10 µL of Matrigel (Corning, New York USA) was used to coat the base of the lower chamber of a 15 μ-3D well plate (Ibidi, Germany Cat:89646). The plate was spun at 400 × *g* for 30 s before the Matrigel was allowed to set at 37 °C for 30 min. HUtMECs were seeded at 1 × 10^4^ cells/well in 70 μL of complete media containing either: vehicle, 25, 50, 100 nmol/L s(P)RR-Flag, or 10 ng/mL TNF-α (*n* = 4 independent experiments performed in technical triplicate). Cells were incubated in each treatment for 6 h at 37 °C, before imaging took place using a Nikon eclipse Ti microscope (Nikon, Japan). Each well (4 images/well, approximately 0.34 cm^2^ per well) was analysed using the angiogenesis analyser plug-in [26] in the ImageJ analysis software (Fuji) [27].

### HUtMEC adhesion assay

Isolated PBMCs were cultured in Calcein (20 μg/mL; Invitrogen) for 45 min at 37 °C in serum-free Roswell Park Memorial Institute (RPMI, Gibco) medium, before being cultured in RPMI supplemented with 10% FBS.

Complete EBM-2 media containing 1 × 10^4^ HUtMEC cells/100 μL, was seeded into each well of a 96-well plate and allowed to attach for 24 h. Each well was then incubated with media containing vehicle, s(P)RR-Flag or TNF-α as above, for 24 h, before being incubated with 7.5 × 10^3^ labelled PBMCs/well for 45 min at 37 °C in serum-free RPMI (Hyclone, Utah USA) (*N* = 3 independent experiments performed in technical triplicate). After PBMC incubation, wells were washed with 100 μL of PBS before being reseeded in complete EBM-2 media for imaging using a Cytation microscope (Biotech, Vermont, United States). Acquired images (4 images/well, approximately 0.34 cm^2^ per well) were analysed utilising ImageJ [27] and the “cell counting” plug-in [27].

### Total nitric oxide level assay

Total nitrate/nitrite levels were assessed in conditioned HUtMEC culture media via Nitric oxide assay kit (ab65328; Abcam) according to the manufacturer’s instructions. A SPECTROstar^Nano^ micro-plate reader was utilised to measure the optical density at 540 nm. Sample dilution buffer was used as a blank correction to correct for background intensity. The intra-assay coefficient of variance (CV) was 4.57% and the inter-assay CV was 3.63%.

### Injection of adenovirus expressing s(P)RR into pregnant rats

When in oestrus, female rats were housed with stud males overnight. The presence of sperm in the vaginal canal was an indication of successful mating and was considered day 1 of pregnancy. On day 8 of pregnancy, rats were anaesthetised with 2% isoflurane in oxygen (Univentor 400, Agnthos, AB, Lidingö, Sweden) and maintained at 1% isoflurane in oxygen, via inhalation. Once anaesthetised, rats were placed on a heating pad (43 °C), and a single bolus dose of adenovirus was injected into the tail vein. Rats were injected with 1 × 10^9^ PFU of adenovirus expressing s(P)RR (+ADV) or an empty adenovirus (-ADV) produced by ViraQuest Inc (Iowa, USA). Dosing was based on pilot studies (Supplementary Figure 3). The adenovirus vector Ad-CMV-s(P)RR-eGFP (NP_005756, 4.0 × 10^10^ PFU, concentration of 1.1 × 10^12^ pts/ml, Lot number: 30858) contained the rodent s(P)RR cDNA encoding the extracellular domain of the (P)RR and produced a final product of 6984 bp. The adenovirus vector Ad-EMPTY-eGFP (4.0 × 10^10^ PFU, concentration of 1.1 × 10^12^ pts/ml, Lot number: 30858) contained a nonsense cDNA sequence. Rats were monitored daily until post-mortem (*N* = 6–7 dams per treatment group).

### Blood pressure measurement

Blood pressure was measured in conscious rats using non-invasive tail-cuff plethysmography (Kent Scientific) as described previously by our group [28]. Rats underwent a five-stage acclimation process over five days prior to initial measurements according to the manufacturer instructions. Blood pressure was measured prior to pregnancy (non-pregnant) and then again on days 15 and 17 of pregnancy. Measurements were taken between 0800 and 0900 hours, with each testing protocol allowing rats 5 min to acclimate to the system, followed by 10 acclimation cycles and then 20 test cycles per rat.

### Tissue collection and post-mortem

Urine was collected the day before mating between 0800 and 0900 hours. Female rats were placed on a sterile hard surface covered in clean glad wrap until urination. Urine was then collected via pipette and stored at −80 °C.

On day 18 of pregnancy, rats were anaesthetised by 4% isoflurane in oxygen so that a blood sample (~8 mL) could be collected by cardiac puncture. Plasma samples were stored at −80 °C for subsequent measurements. Rats were then humanely euthanised by removal of the heart after opening the chest cavity. Urine was collected via needle directly from the bladder (~0.2 mL). The uterine horns were dissected from the abdominal cavity and each fetus was separated from its placenta and weighed. The rat’s left kidney and the mesenteric artery were collected and immediately placed in ice-cold Krebs (physiological saline solution: PSS mmol/L: NaCl 120, KCl 5, MgSO_4_ 1.2, KH_2_PO_4_ 1.2, NaHCO_3_ 25, D-glucose 11.1, and CaCl_2_ 2.5, and bubbled with carbogen (95% O_2_ and 5% CO_2_)) before isolation of the interlobar renal and small mesenteric arteries (described below).

### Isolation of arteries and assessment of vascular reactivity

Interlobar renal and small mesenteric arteries (third-order branches of the superior mesenteric artery) were isolated (described above), cleared of fat and loose connective tissue, and cut into 2 mm rings. After arteries were mounted on the myograph (model 610M; Danish Myo Technology, Aarhus, Denmark), they were allowed to stabilise for 15 min before normalisation, as described previously [29]. Vascular reactivity was measured in real-time using LabChart software (ADInstruments, NSW, Australia). After normalisation, arteries were contracted with high potassium physiological saline solution (KPSS; K^+^ = 100 mM, isosmotic replacement of Na^+^ with K^+^). Subsequently, the integrity of the endothelium was determined by submaximally preconstricting arteries with the α_1_-adrenoceptor agonist, phenylephrine (mesenteric arteries) or a combination of phenylephrine and the thromboxane A2 mimetic U46619 (renal arteries). Then, the endothelium-dependent dilator acetylcholine (ACh, 10^−^^5^ M) was added at 50–70% of the vessel’s maximum constriction to induce relaxation and to test whether the endothelium was functional. Arteries with >80% relaxation were deemed suitable for further analysis (*N* = 6–7 arteries per treatment group).

To assess endothelium-dependent and endothelium-independent vasodilator function, both renal and mesenteric arteries were pre-contracted to 50–70% of maximum KPSS contraction using phenylephrine (0.1–3 μm), and concentration-response curves to the endothelium-dependent agonists acetylcholine (ACh, 10^−^^10^–10^−^^5^ M) or the endothelium-independent agonist sodium nitroprusside (SNP, 10^−^^10^–10^−^^5^ M) were determined [30]. Relaxation was expressed as a percentage of the level of preconstriction.

To examine contraction, arteries were exposed to increasing concentrations of various vasoconstrictors including PE (10^−^^9^–10^−^^4.5^ M), U46619 (10^−^^10^–10^−^^6.5^ M) or endothelin-1 (ET-1, 10^−^^10^–10^−^^7^ M). Contractions were expressed as a percentage of the contraction evoked by 100 mM KPSS. All vascular drugs were purchased from Sigma-Aldrich and were dissolved in distilled water.

### Rat enzyme-linked immunosorbent assays (ELISAs)

Commercial ELISA kits were used to measure s(P)RR (IBL-America, IBJP27782) and leptin (Abcam, ab229891) in maternal plasma following manufacturer’s instructions

Protein in rat urine was determined by measuring concentrations of albumin and creatinine in maternal urine samples using two commercially available ELISA kits (ABCAM Australia Pty Ltd; albumin, ab108789; creatinine, ab65340) according to manufacturer instructions (Supplementary Table 2). A SPECTROstar^Nano^ micro-plate reader was utilised to measure the optical density at 450 nm for each sample. Sample dilution buffer was used as a blank correction to correct for background intensity.

### Statistical analyses

All statistical analyses were performed using GraphPad Prism 10 (GraphPad Software, Inc) and a *P* value of <0.05 was considered statistically significant.

Data from in vitro cell culture experiments was derived from *n* = 3–4 independent experiments performed in technical triplicate. The distribution of in vitro data (parametric vs non-parametric) was determined using a Shapiro–Wilk test. Parametric data (ICAM-1 mRNA, nitric oxide levels, ET-1 protein, IL-6 protein, and TNF-α protein) were analysed using a one-way ANOVA with Dunnett’s multiple comparisons test. Non-parametric data (VCAM-1 mRNA and protein, ET-1 mRNA) were analysed using a Kruskal–Wallis test with Dunn’s multiple comparisons test. Additionally, in vitro angiogenesis (*n* = 4) and adhesion assay (*n* = 3) data were analysed by a one-way ANOVA with Dunnett’s multiple comparisons test.

Data from the in vivo rat experiments (*n* = 6–7 dams per treatment group) were analysed as follows. The distribution of in vivo data (parametric vs non-parametric) was determined using a Shapiro–Wilk test. Maternal blood pressure was assessed using a two-way ANOVA with mixed-effects analysis. All fetal/placental characteristics are presented as litter average. Parametric data (fetal weight, placental weight, number of reabsorptions, fetal/placental weight ratio, maternal urinary creatinine, and maternal urinary albumin) were analysed using an unpaired *t*-test. Non-parametric data (litter size, maternal leptin, and maternal s(P)RR) were analysed using an unpaired *t*-test with Mann–Whitney corrections.

All vascular sensitivity analyses were performed as follows. Normality (parametric vs non-parametric) was determined using a Shapiro–Wilk test. Non-parametric data (mesenteric artery SNP_Rmax_, PE _pEC50_, U46619 _pEC50_, and renal artery ACh_Rmax_) were analysed using a paired t-test with Wilcoxon matched-pairs signed rank correction. Parametric data (remaining arterial sensitivity assessments) were analysed by using a standard paired t-test.

## Results

### Treatment with soluble (P)RR increased markers of endothelial dysfunction in vitro

**Fig. 1:**
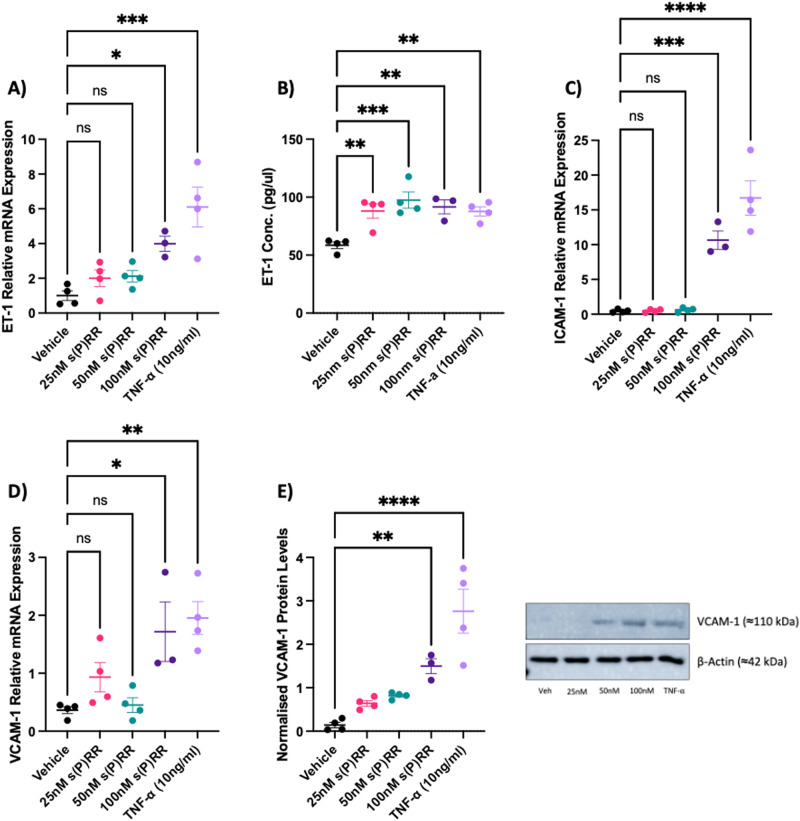
Soluble prorenin receptor (s(P)RR) induced endothelial dysfunction in HUtMECs. HUtMECs were treated with increasing doses of an s(P)RR-flag peptide (25, 50, and 100 nM), 10 ng/mL of TNF-α (positive control) or vehicle (media alone) and cultured for 24 h. Both s(P)RR and TNF-α treatment successfully increased **A** ET-1 mRNA expression, **B** ET-1 protein levels and **C** ICAM-1 mRNA expression. Additionally, s(P)RR and TNF-α treatment successfully increased **D** VCAM-1 mRNA expression and **E** VCAM-1 protein levels. *P < 0.05, **P < 0.005, ***P < 0.0005, ****P < 0.0001 indicate a significant difference between the vehicle control and the respective treatment groups. β-actin was used as a loading control in the representative immunoblot. Shapiro–Wilk normality tests were performed for all statistical analyses. ELISA and qPCR data was analysed through an ordinary one-way ANOVA with Dunnett’s Multiple comparisons test. Immunoblot data was analysed by one-way ANOVA with Kruskal–Wallis: Dunn’s Multiple comparisons test. Data sets are presented as the mean ± SEM. *N* = 3–4 experiments conducted in technical triplicate

Treatment of HUtMECs with recombinant s(P)RR or TNF-α significantly increased markers of endothelial dysfunction at both the mRNA and protein level compared with the media-only control. Recombinant s(P)RR significantly increased ET-1 mRNA (100 nM s(P)RR: *P* = 0.025; and TNF-α: *P* = 0.002, Fig. 1A) and protein secretion (25 nM s(P)RR: *P* = 0.003; 50 nM s(P)RR: *P* = 0.001; 100 nM s(P)RR: *P* = 0.003; and TNF-α: *P* = 0.003, Fig. 1B), as well as ICAM-1 mRNA (100 nM s(P)RR: *P* = 0.001; and TNF-α: *P* < 0.001, Fig. 1C) with s(P)RR treatment compared with the vehicle only control. Additionally, VCAM-1 mRNA (100 nM s(P)RR: *P* = 0.018; and TNF-α: *P* = 0.002, Fig. 1D) and protein secretion (100 nM s(P)RR: *P* < 0.001; and TNF-α: *P* < 0.001, Fig. 1E) were increased compared to the vehicle control.

### Recombinant s(P)RR impairs HUtMEC arterial tube formation in vitro

**Fig. 2:**
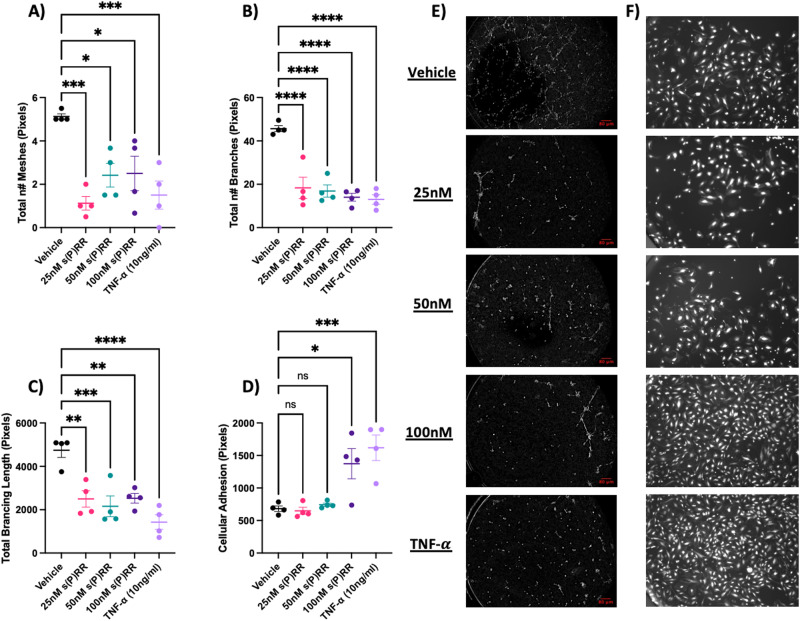
Soluble prorenin receptor (s(P)RR) impairs vascular formation and alters endothelial adhesion. HUtMECs were treated with increasing doses of an s(P)RR-flag peptide, 10 ng/mL of TNF-α or vehicle control (media alone) and cultured for 24 h on Matrigel to facilitate tube formation. Treatment with both s(P)RR and TNF-α impaired arterial formation with reduced **A** total number of a meshes, **B** total number of branches, and **C** total branching length. In subsequent experiments, treated HUtMECs were cultured in the presence of peripheral blood mononuclear cells. **D** s(P)RR treatment enhanced endothelial adhesion. **E** Representative images of tube formation assay between different treatment groups of analysis shown in (**A**, **B**, **C**). **F** Representative images of endothelial adhesion assay between different treatment groups of analysis shown in (**D**). *P < 0.05, **P < 0.005, ***P < 0.0005, ****P < 0.0001 indicate a significant difference between the vehicle control and the treatment groups. Shapiro–Wilk normality tests were performed for all statistical analyses. Data were analysed by an ordinary one-way ANOVA with Dunnett’s multiple comparisons test and are presented as mean ± SEM. *N* = 3–4 experiments conducted in technical triplicate

The angiogenic capacity of HUtMECs treated with recombinant s(P)RR was assessed through an endothelial tube formation assay. Treatment of HUtMECs with either s(P)RR or TNF-α significantly impacted measures of angiogenic capacity when compared to the media-only control. The area enclosed within arterial formations (meshes) was significantly reduced by all treatments (25 nM s(P)RR: *P* = 0.001; 50 nM s(P)RR: *P* = 0.010; 100 nM s(P)RR: *P* = 0.013; and TNF-α: *P* = 0.001 Fig. 2A). Additionally, all treatments significantly decreased total branch number (25 nM s(P)RR: *P* < 0.001; 50 nM s(P)RR: *P* < 0.001; 100 nM s(P)RR: *P* < 0.001; and TNF-α: *P* < 0.001 Fig. 2B) and length (25 nM s(P)RR: *P* = 0.002; 50 nM s(P)RR: *P* = 0.001; 100 nM s(P)RR: *P* = 0.002; and TNF-α: *P* < 0.001 Fig. 2C). Representative images of HUtMEC tube formation assays following the various treatments are shown in Fig. 2E.

### Recombinant s(P)RR alters endothelial vascular adhesion in vitro

Cellular adherence of HUtMECs treated with recombinant s(P)RR was assessed via co-culture with PBMCs. HUtMECs treated with either 100 nM s(P)RR or TNF-α significantly increased adherence of PBMCs (100 nM s(P)RR: *P* = 0.011; and TNF-α: *P* = 0.001 Fig. 2D) when compared to vehicle only control. Representative images of the adherence assay between treatment options are shown in Fig. 2F.

### Recombinant s(P)RR treatment did not affect markers of inflammation in vitro

**Fig. 3:**
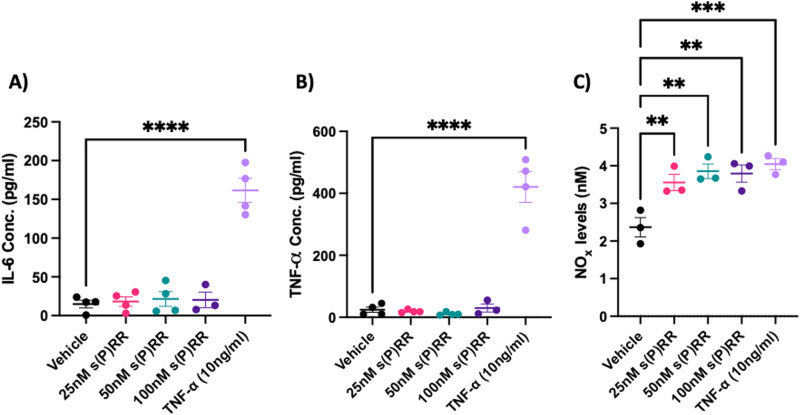
The soluble prorenin receptor (s(P)RR) does not increase markers of inflammation. HUtMECs were treated with increasing doses of an s(P)RR-flag peptide, 10 ng/mL of TNF-α or vehicle control (media alone) and cultured for 24 h. Conditioned media was assessed for markers of inflammation. Treatment with TNF-α significantly increased **A** IL-6, and **B** TNF-α concentration compared with the vehicle control. Soluble (P)RR treatment had no effect. Total nitric oxide (NO) was determined from total nitrate and nitrite. All concentrations of s(P)RR and TNF-α significantly increased **C** nitric oxide levels compared with the vehicle control. **P < 0.005, ***P < 0.0005, ****P < 0.0001 indicate a significant difference between the vehicle control and the treatment groups. Shapiro–Wilk normality tests were performed for all statistical analyses. Data were analysed by an ordinary one-way ANOVA with Dunnett’s multiple comparisons test and are presented as mean ± SEM. *N* = 3–4 experiments conducted in technical triplicate

Inflammatory markers were assessed in the HUtMEC supernatant in response to s(P)RR or TNF-α treatment. Treatment of HUtMECs with TNF-α, but not s(P)RR, significantly increased IL-6 (100 nM s(P)RR: *P* = 0.953; TNF-α: *P* < 0.001 Fig. 3A) and TNF-α (100 nM s(P)RR: *P* = 0.985; TNF-α: *P* < 0.001 Fig. 3B) protein levels when compared with the vehicle control. All doses of recombinant s(P)RR and TNF-α treatment increased total nitric oxide levels (25 nM s(P)RR: *P* = 0.009; 50 nM s(P)RR: *P* = 0.002; 100 nM s(P)RR: *P* = 0.003; and TNF-α: *P* = 0.001 Fig. 3C) when compared with the vehicle control.

### Positive adenovirus-treated rats displayed increased circulating s(P)RR

Maternal serum was collected from rats at day 18 (D18) of pregnancy, and serological levels of s(P)RR were assessed. Pregnant rats treated with the s(P)RR overexpressing adenovirus (+ADV) displayed significantly increased serum s(P)RR levels (*P* = 0.001; Supplementary Fig. 2), when compared with the control adenovirus-treated group (−ADV).

### Overexpression of maternal s(P)RR reduced fetal growth

At D18 of pregnancy, rats were euthanised and the uterine horns and fetuses collected to assess both litter size and fetal reabsorptions throughout gestation (Table 1). No differences were observed between the −ADV and +ADV groups for litter size (*P* = 0.417) or the number of fetal reabsorptions (*P* = 0.217).

**Table 1:** Fetal outcomes

	−ADV	+ADV	*P Values*
	*n* = *6*	*n* = *7*	
Pregnancy success			
Litter Size	11.50 ± 1.02	10.71 ± 0.71	0.417
Reabsorptions per pregnancy	0.67 ± 0.33	1.71 ± 0.68	0.217
Pregnancy weights (g)			
Fetal	0.90 ± 0.09	**0.56** ± 0.06*	**0.008**
Placental	0.33 ± 0.01	0.33 ± 0.02	0.761
Fetal/Placental weight ratio	2.38 ± 0.29	1.92 ± 0.16	0.183

Data are presented as mean ± SEM. Data for litter size and fetal weight were analysed by an unpaired *t*-test with Mann–Whitney test. Data for pregnancy reabsorptions, placental weight, and fetal/placental weightratio were analysed by a standard unpaired *t*-test. Shapiro–Wilk test was used to determine normality

*Values in bold font denotes a significant difference between groups (*P* ≤ 0.005)

Fetuses and their corresponding placental tissue were weighed at D18 (Table 1). The average fetal weight per litter was significantly lower in ADV+ compared to the ADV− groups (*P* = 0.008), with no changes observed in placental weight (*P* = 0.761) or fetal-placental weight ratio (*P* = 0.183) between groups.

### Pregnant rats with increased maternal s(P)RR displayed increased blood pressure

**Fig. 4:**
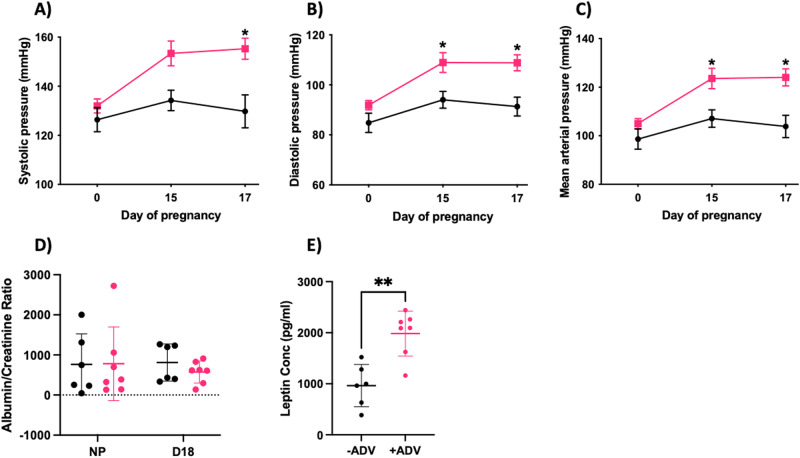
Rats overexpressing s(P)RR produce a preeclamptic-like phenotype. Rats were treated with either a control adenovirus (-ADV) or one containing s(P)RR (+ADV). Rats in the +ADV group displayed **A** elevated systolic blood pressure at pregnancy day 17 with **B** increased diastolic blood pressure at both days 15 and 17. When compared together, **C** +ADV treatment increased mean arterial pressure at both days 15 and 17. Collected rat urine was assessed for urinary albumin and creatinine secretion with **D** no changes in the albumin:creatinine ratio before pregnancy (NP) and at term pregnancy (D18) between treatment groups. Additionally, maternal rat serum was collected and examined for leptin concentration and showed **E** significantly increased leptin concentration in the +ADV treatment compared with the −ADV group. *P < 0.05, ***P < 0.0005, ****P < 0.0001, indicate a significant difference between the −ADV and the +ADV treatment groups. Shapiro–Wilk normality tests were performed for all statistical analyses. Blood pressure data were analysed using a two-way ANOVA with mixed-effects analysis. Maternal urinary protein and serum protein were analysed using an unpaired *t*-test with Mann–Whitney test. All data are presented as mean ± SEM. *N* = 6–7 Litters

Maternal blood pressure was measured before pregnancy at key time points throughout gestation and compared between −ADV and +ADV groups. At day 17 of pregnancy, maternal systolic blood pressure was elevated in the +ADV group compared with the −ADV group (*P* = 0.001; Fig. 4A). In conjunction, both day 15 and 17 maternal diastolic blood pressures were elevated with s(P)RR overexpression (+ADV: D15, *P* = 0.039; D17, *P* = 0.010; Fig. 4B) compared with the −ADV group. Furthermore, mean arterial pressure was also elevated at days 15 and 17 in the s(P)RR overexpressing treatment group (+ADV; D15, *P* = 0.031; D17, *P* = 0.012; Fig. 4C) when compared with the −ADV group.

### In pregnant rats, elevated maternal s(P)RR does not induce proteinuria

Urine was obtained before pregnancy (NP) and at D18, through spot urine collections. Concentrations of urinary albumin and creatinine were determined and the albumin: creatinine ratio determined. No significant changes were observed between the treatment groups either before pregnancy or at term (NP: *P* = 0.388; D18: *P* = 0.445; Fig. 4D).

### Maternal serum leptin levels increased in response to elevated s(P)RR in pregnant rats

Maternal serum was collected at D18 from pregnant rats, and serological levels of leptin were assessed. Maternal soluble (P)RR overexpression (+ADV) significantly increased maternal serum leptin levels (*P* = 0.001; Fig. 4E), when compared with the −ADV group.

### Elevated maternal s(P)RR impairs renal artery sensitivity in pregnant rats

At term pregnancy, both the mesenteric and renal artery of rats were assessed via wire myography to determine arterial sensitivity (Table 2). Elevated s(P)RR levels were not associated with an alteration in mesenteric artery responses to ACh, SNP, Phenylephrine, U46619 or ET-1. Renal arteries however, showed decreased sensitivity to the endothelium-dependent dilator ACh in the +ADV group (pEC_50_, *P* = 0.028; Table 2), indicative of endothelial dysfunction. The renal artery did not demonstrate any other altered response to any other drug treatment (Table 2).

**Table 2:** Rat arterial sensitivity

	−ADV		+ADV		*P Values*	
	*n* = *6*		*n* = *7*			
	pEC_50_	*R*_max_	pEC_50_	*R*_max_	pEC_50_	*R*_max_
Mesenteric artery						
ACh	7.83 ± 0.23	97.40 ± 1.59	7.90 ± 0.06	99.21 ± 0.52	0.725	0.294
SNP	7.59 ± 0.21	95.47 ± 1.47	7.81 ± 0.12	96.73 ± 1.21	0.276	0.063
Phenylephrine	5.72 ± 0.09	133.0 ± 3.30	5.61 ± 0.03	137.7 ± 5.12	0.219	0.479
U46619	7.27 ± 0.14	118.5 ± 6.42	7.39 ± 0.09	128.4 ± 4.06	0.219	0.302
ET-1	8.06 ± 0.17	129.8 ± 6.92	8.21 ± 0.11	137.3 ± 4.82	0.614	0.063
Renal artery						
ACh	7.47 ± 0.27	81.19 ± 4.83	**6.68** ± 0.17*	73.48 ± 5.51	**0.028**	0.438
SNP	7.90 ± 0.22	92.04 ± 3.79	7.64 ± 0.13	93.41 ± 1.60	0.152	0.862
Phenylephrine	6.22 ± 0.21	145.6 ± 3.83	6.21 ± 0.09	146.5 ± 3.87	0.942	0.955
U46619	7.43 ± 0.14	127.5 ± 3.29	7.65 ± 0.14	128.3 ± 3.82	0.286	0.974
ET-1	8.24 ± 0.11	137.1 ± 3.75	11.24 ± 3.01	147.3 ± 4.35	0.292	0.081

Data are presented as mean ± SEM. Mesenteric artery data SNP _Rmax_, Phenylephrine _pEC50_, U46619 _pEC50_, and Renal artery data ACh _Rmax_ were analysed by a paired *t*-test with the Wilcoxon matched-pairs signed rank test. Remaining data sets were analysed by a standard paired *t*-test. Shapiro–Wilk test was used to determine normality

*and values in bold font denotes a significant difference between groups (*P* ≤ 0.05)

## Discussion

In the present study, we interrogated the role that elevated s(P)RR levels play in promoting a preeclamptic-like phenotype using both an in vitro and an in vivo model. Our in vitro studies showed that s(P)RR impaired vascular endothelial function in HUtMECs. Additionally, we are the first to show that overexpression of s(P)RR in pregnant rats produces a preeclampsia-like phenotype with elevated maternal blood pressure, altered renal artery sensitivity, and restricted fetal growth.

Treatment of HUtMEC cells with recombinant s(P)RR (Fig. 1) resulted in increased levels of endothelial dysfunction markers; VCAM-1, ICAM-1, and ET-1, suggesting that elevated s(P)RR has the potential to cause systemic maternal endothelial dysfunction. Recently, Mishima et al. highlighted a connection between increased placental (P)RR and elevated plasma ET-1 secretion in a RUPP mouse model of preeclampsia [31]. Collectively, these data suggest a positive correlation with elevated (P)RR/s(P)RR and endothelial dysfunction. Moreover, two separate studies have shown that the s(P)RR acts as a mediator of Ang II-induced hypertension in mice [32, 33]. HUtMECs treated with recombinant s(P)RR had reduced angiogenic capacity and altered vascular adhesion properties (Fig. 2). As such, s(P)RR may be promoting hypertension and altered arterial function, key characteristics of preeclamptic pregnancies.

Recent studies have shown that human renal epithelial cells treated with s(P)RR, can stimulate the production/release of pro-inflammatory factors including IL-6, IL-8, and transforming growth factor β1 [34]. Yang et al. showed that upregulating (P)RR in human umbilical vein endothelial cells (HUVECs), increased inflammatory markers (IL-1β, IL-6, and ICAM-1) via uric acid production [35]. More specifically, Fu et al. has highlighted that the direct interaction between s(P)RR and the AT_1_R in HUVECs, promotes the upregulation of IL-6, IL-8, VCAM-1, and ICAM-1 mRNA expression [12]. Together, these data suggest that s(P)RR contributes to the endothelial inflammatory phenotype as seen in preeclamptic pregnancies. However, we observed no increases in the inflammatory markers; IL-6 and TNF-α, in HUtMECs when treated with recombinant s(P)RR. Therefore, our data suggests that s(P)RR-induced endothelial dysfunction in HUtMECs is not due to induction of an inflammatory phenotype.

Additionally, treatment of HUtMECs with recombinant s(P)RR increased anti-inflammatory nitric oxide production (Fig. 3). Again, this is in direct contrast with data produced by Fu et al. who showed that direct binding of the s(P)RR with AT_1_R led to the suppression of nitric oxide production by endothelial cells [12]. The difference between our results could be due to the difference in cell lines used. Altered expression of AT_1_R may result in an imbalance in the RAS cascade that favours the Angiotensin-(1-7)/Mas receptor arm. The Ang 1-7/Mas receptor arm of the RAS cascade promotes nitric oxide production, while decreasing inflammatory mediator expression [36]. This could account for the unchanged IL-6 and TNF-α expression and increased NO production seen in our study and indicates that some of the actions of s(P)RR may be via stimulation of the Ang-(1-7)/Mas receptor pathway rather than the Angiotensin II/AT_1_R pathway. Further studies are however required to fully elucidate the mechanism by which s(P)RR produces these effects.

We are the first to examine the effects of s(P)RR overexpression in an in vivo pregnant rat model. Adenovirus overexpression of s(P)RR in pregnant rats resulted in reduced fetal weight (Table 1), maternal hypertension (Fig. 4) and altered renal artery sensitivity to ACh (Table 2) compared with -ADV controls, all key clinical signs of preeclampsia [37]. Interestingly, ACh-induced vasodilation is endothelium dependent, while the remaining drug treatments (SNP, Phenylephrine, U46619, or ET-1) are smooth muscle dependent [38]. Thereby elevated circulating s(P)RR may only effect endothelial cells in the interlobar arteries, and the smooth muscle around the renal and mesenteric arteries may not have been affected in this in vivo model. Additionally, our rat model displayed increased serum leptin levels in animals overexpressing s(P)RR, compared with control animals (Fig. 4). Circulating leptin levels are known to be increased in preeclamptic pregnancies in comparison with normotensive pregnancies [19]. A study by Gatineau et al. has highlighted that high fat-fed male mice with elevated s(P)RR have increased plasma leptin levels [20], potentially impairing baroreflex sensitivity and increasing systolic blood pressure. Furthermore, previous human cohort studies have also highlighted that high circulating s(P)RR levels during early pregnancy are associated with elevated blood pressure at term [39]. Similar results were found in our study where elevated circulating s(P)RR levels from mid-gestation in rats, produced marked hypertension (increased systolic, diastolic, and mean arterial blood pressure) in late pregnancy (Fig. 4), further implicating s(P)RR in the development of hypertensive disorders in pregnancy.

While overexpression of s(P)RR in vivo resulted in the key hallmarks of preeclampsia (hypertension, foetal weight reduction, and increased serum leptin), proteinuria was not observed (Fig. 4). This is again in contrast to Fang et al. who showed that the s(P)RR could produce pro-inflammatory factors (IL-6 and IL-8) in renal epithelial cells [34], indicating a potential role of the s(P)RR in renal inflammation. However, in our study, elevated s(P)RR in rats was associated with decreased renal artery sensitivity to the relaxation-inducing effects of acetylcholine, while no changes were observed in mesenteric arterial sensitivity (Table 2). These data suggest that s(P)RR alone, may not be sufficient to produce renal injury in vivo, but can promote renal vascular dysfunction [12].

Recently, Li et al. developed an antagonistic peptide specific for the (P)RR, PRO20, which inhibits the catalytic capacity of both renin and prorenin bound to the (P)RR [40]. The handle region of the peptide inhibits the confirmational change and nonproteolytic activation of prorenin that occurs when binding to the (P)RR. Interestingly, in a mouse model of 5/6 nephrectomy, increased urinary/renal levels of renin activity, angiotensinogen, and Angiotensin II, were attenuated by antagonism of the (P)RR with PRO20. As such, PRO20 therapy may ameliorate the renal complications arising from elevated maternal s(P)RR. A very recent study has also shown that targeting the s(P)RR may be a novel therapeutic pathway for the treatment of preeclampsia. Mice treated with the handle region peptide (HRP), another (P)RR inhibitor, reduced maternal blood pressure and proteinuria, inhibited endothelin-1 production and improved fetal weights in a mouse model of preeclampsia (RUPP) [31].

In this study, two different models were used to assess the effects of s(P)RR overexpression both in vitro and in vivo. These models together provide strong evidence that elevated s(P)RR levels impair endothelial function and promote hypertension. While recombinant s(P)RR altered markers of endothelial function in vitro, the physiological relevance of the s(P)RR concentrations used in this study is a potential limitation. Several in vitro mechanistic studies of s(P)RR function have used treatment concentrations between 10 and 100 nM [12, 41–43] to determine the functional role s(P)RR pathways in pathological conditions. However, studies assessing maternal circulating s(P)RR levels in preeclamptic women have shown that concentrations are between 30 and 40 ng/ml (approximately 1.1–1.5 nM) [39, 44]. This study utilised concentrations of between 25 and 100 nM of recombinant human s(P)RR (approximately 25–100-fold higher than levels seen in the maternal circulation of preeclamptic patients). As such, changes in endothelial function produced in this model may not be representative of human in vivo assessment. In contrast, levels of plasma s(P)RR measures within this rat model displayed a 3.6-fold increase between the +ADV and −ADV groups. In humans, s(P)RR levels are elevated by approximately 1.6-fold in patients with preeclampsia (30–40 ng/ml) compared with normotensive pregnancies (25 ng/ml) [8]. As such, the rat model highlighted in this study may be more physiologically relevant.

Next, the differences in HUVEC and HUtMEC cellular responses to s(P)RR treatment needs to be addressed. Within HUtMECs, s(P)RR increased NO production with no marked increases in inflammatory factors (IL-6 and TNF-α). Conversely, s(P)RR decreased NO production while increasing IL-6 and IL-8 inflammatory factors [12]. The underlying reason for these fundamental differences could be due to the differences in each type of cell line. HUtMECs are a uterine microvascular endothelial cell line and thus are more representative of maternal vascular environment than HUVECs. HUVECs are a fetal vascular cell line and are more reflective of fetal vascular dysfunction [45]. In our study, we have shown that when treated with TNF-α or preeclamptic serum, HUtMECs respond in a similar manner to that reported in HUVECs. That is, they increase their expression of ET-1, ICAM, VCAM, IL-6 and can alter total nitric oxide levels [46–49]. However, no studies have compared the response of these endothelial cell lines to s(P)RR treatment. As such, the differences in the inflammatory phenotype produced by s(P)RR remains to be fully explored and needs to be addressed in the future.

To fully understand the role s(P)RR plays in preeclamptic pregnancy, understanding the relationship s(P)RR plays with key pathogenetic factors of PE is necessary. As highlighted previously, the s(P)RR can directly interact with the AT_1_R, disputing the notion that Ang II is the sole activating ligand for the AT_1_R [12]. Considering that Ang II/AT_1_R signalling promotes the secretion of anti-angiogenic factors including sFlt-1 and sEng [50, 51]. Taken together, s(P)RR signalling via the AT_1_R may directly influence the secretion of anti-angiogenic factors such as sFlt-1 throughout pregnancy, however future experiments are required to fully explore this relationship.

To conclude, we have shown that the s(P)RR is involved in endothelial dysfunction and hypertension, key hallmarks of preeclampsia. Treatment of HUtMECs with recombinant s(P)RR increased the expression of key endothelial function markers; VCAM-1, and ET-1, reduced their angiogenic capacity and increased vascular cellular adhesion, highlighting the potential for s(P)RR to modulate vascular function and inflammation directly or indirectly. We have also confirmed that elevated maternal circulating s(P)RR during pregnancy results in elevated blood pressure in vivo. Additionally, elevated circulating s(P)RR levels altered renal vascular reactivity without resulting in proteinuria, highlighting that s(P)RR alone may not be sufficient to produce kidney dysfunction, a key symptom of preeclampsia. Therefore, these data suggest an important role for elevated maternal circulating s(P)RR in promoting a preeclamptic phenotype.

### Supplementary information

Supplementary Table 1

Supplementary Table 2

Supplementary Figure 1

Supplementary Figure 2

Supplementary Figure 3

## Data Availability

The data used to support the findings of this study are included within the article.
