# Supplementary material for: The soluble (pro)renin receptor promotes a preeclampsia-like phenotype both in vitro and in vivo
Source: Hypertens Res. 2024 Apr 11;47(6):1627–41. doi: 10.1038/s41440-024-01678-8 (PMC11150152; doi:10.1038/s41440-024-01678-8)
Supplement: Supplementary file 1 — Supplementary Table 1 [file 41440_2024_1678_MOESM1_ESM.doc]

**Supplementary Table 1: Primer details for qPCR**

| **Gene** | **GenBank Accession #** | **Primer Sequence (5' to 3')** | **Concentration (nm)** |
| --- | --- | --- | --- |
| *ACTB* | NM_001101 | Fwd: CGCGAGAAGATGACCCAGAT | 1000 |
|  |  | Rev: GAGTCCATCACGATGCCAGT |  |
| *B2M* | NM_004048 | Fwd: AAGGACTGGTCTTTCTATCTC | 600 |
|  |  | Rev: GATCCCACTTAACTATCTTGG |  |
| *YWHAZ* | NM_003406 | Fwd: CCTGCATGAAGTCTGTAACTGAG | 100 |
|  |  | Rev: GACCTACGGGCTCCTACAACA |  |
| *VCAM-1* | NM_00107 | Fwd: ACTTGATGTTCAAGGAAGAG | 400 |
|  |  | Rev: TCCAGTTGAACATATCAAGC |  |
| *ICAM-1* | NM_000201 | Fwd: ACCATCTACAGCTTTCCG | 200 |
|  |  | Rev: TCACACTTCACTGTCACC |  |
| *ET-1* | NM_001416564 | Fwd: CAAGCAGGAAAAGAACTCAG | 400 |
|  |  | Rev: CTGGTTTGTCTTAGGTGTTC |  |

Abbreviations: *Fwd;* forward sequence, *Rev;* reverse sequence, *ACTB;* β-actin, *B2M;* beta-2 macroglobulin*, YWHAZ;* Tyrosine 3-Monooxygenase/Tryptophan 5-Monooxygenase Activation Protein Zeta, VCAM-1; vascular cell adhesion protein 1, ICAM-1; intracellular adhesion molecule 1, ET-1; endothelin 1.
