# Supplementary material for: The soluble (pro)renin receptor promotes a preeclampsia-like phenotype both in vitro and in vivo
Source: Hypertens Res. 2024 Apr 11;47(6):1627–41. doi: 10.1038/s41440-024-01678-8 (PMC11150152; doi:10.1038/s41440-024-01678-8)
Supplement: Supplementary file 2 — Supplementary Table 2 [file 41440_2024_1678_MOESM2_ESM.doc]

**Supplementary Table 2: ELISA details**

| **Target** | **Manufacturer** | **Intra Assay CV%** | **Inter Assay CV%** |  |
| --- | --- | --- | --- | --- |
| *ET-1* | Invitrogen (EIAET1) | 3.14 | 5.73 |  |
|  |  |  |  |  |
| *IL-6* | RnD Systems (DY206-05) | 9.75 | 7.23 |  |
|  |  |  |  |  |
| *TNF-α* | RnD Systems (DY210-05) | 8.76 | 8.94 |  |
|  |  |  |  |  |
| *s(P)RR* | IBL-America (IBLJP27782) | 2.14 | n/a |  |
|  |  |  |  |  |
| *Leptin* | Abcam (ab229891) | 15.93 | n/a |  |
|  |  |  |  |  |
| *Albumin* | Abcam (ab108789) | 1.74 | n/a |  |
|  |  |  |  |  |
| *Creatinine* | Abcam (ab65340) | 2.22 | n/a |  |
|  |  |  |  |  |

Abbreviations: *ET-1; endothelin-1, IL-6; interlukein-6, TNF-α; tumour necrosis factor alpha, s(P)RR; soluble (pro)renin receptor, CV; coefficient of variance.*
