# Supplementary material for: The soluble (pro)renin receptor promotes a preeclampsia-like phenotype both in vitro and in vivo
Source: Hypertens Res. 2024 Apr 11;47(6):1627–41. doi: 10.1038/s41440-024-01678-8 (PMC11150152; doi:10.1038/s41440-024-01678-8)
Supplement: Supplementary file 3 — Supplementary Figure 1 [file 41440_2024_1678_MOESM3_ESM.docx]

*
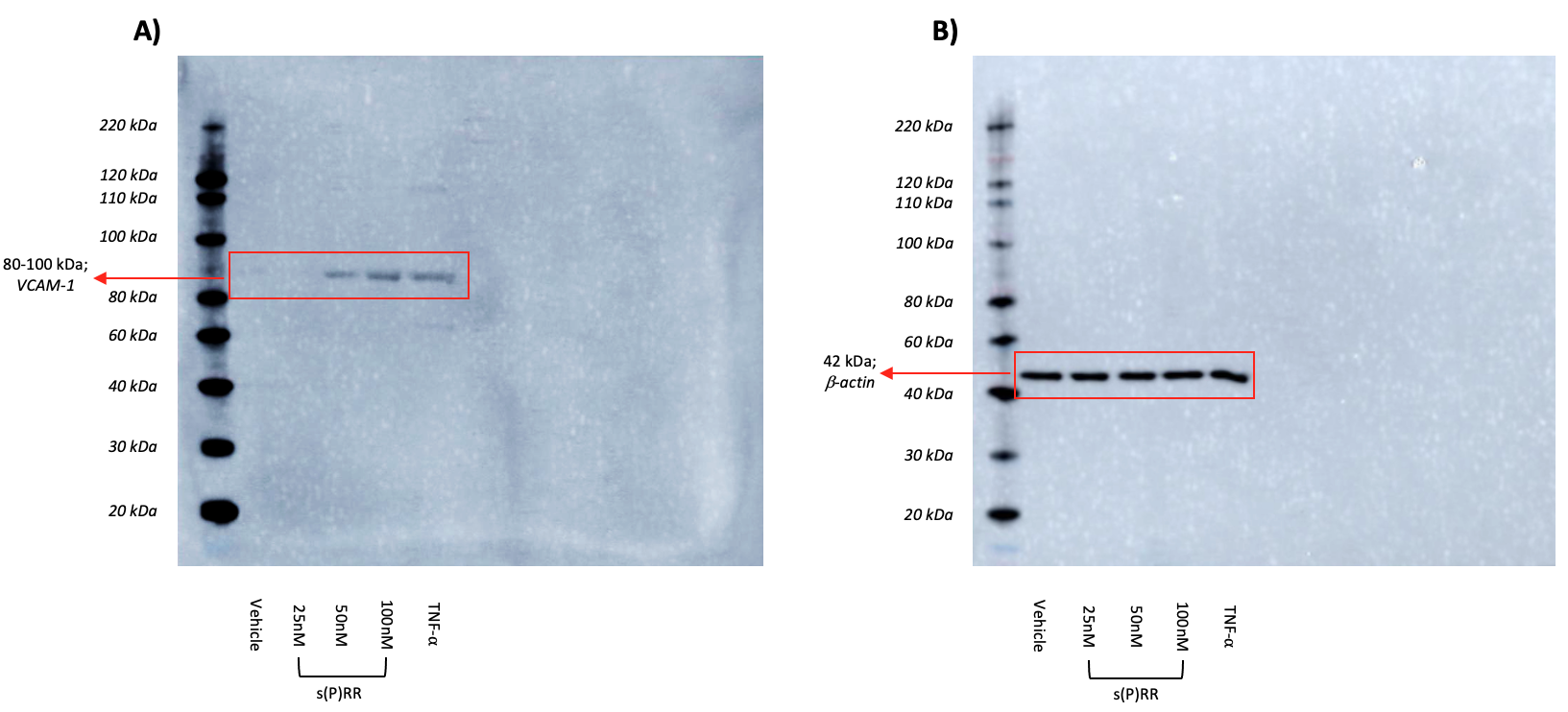
Supplementary Figure 1: Representative full-length immunoblot image of VCAM-1 and β-actin densitometry for HUtMEC cell lysates.* In HUtMECs **A),** VCAM-1 was detected as a clear singular band between 80-100 kDa and **B),** β-actin was also detected as a clear singular band at 42 kDa. Internal control samples are not represented on these immunoblots. **A/B),** are full length blots of the representative figure in Figure 1. The red boxes depict bands shown in the representative blot.
