# Supplementary material for: The soluble (pro)renin receptor promotes a preeclampsia-like phenotype both in vitro and in vivo
Source: Hypertens Res. 2024 Apr 11;47(6):1627–41. doi: 10.1038/s41440-024-01678-8 (PMC11150152; doi:10.1038/s41440-024-01678-8)
Supplement: Supplementary file 4 — Supplementary Figure 2 [file 41440_2024_1678_MOESM4_ESM.docx]

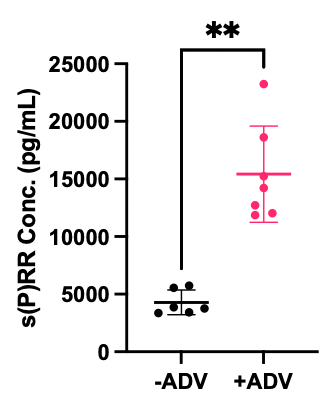
*Supplementary Figure 2:Maternal circulating soluble (pro)renin receptor in adenovirus treated rats.* Rats were treated with either a control adenovirus (-ADV) or one containing s(P)RR (+ADV). Rats in the +ADV group displayed elevated s(P)RR when compared to the -ADV control group. ******:** P<0.0001, indicate a significant difference between the -ADV and the +ADV treatment groups. Shapiro-Wilk normality tests were performed for all statistical analyses. Data were analysed using an unpaired t test with Mann-Whitney test. All data are presented as mean +/- SEM. N=6-7 Litters.
