# Supplementary material for: The soluble (pro)renin receptor promotes a preeclampsia-like phenotype both in vitro and in vivo
Source: Hypertens Res. 2024 Apr 11;47(6):1627–41. doi: 10.1038/s41440-024-01678-8 (PMC11150152; doi:10.1038/s41440-024-01678-8)
Supplement: Supplementary file 5 — Supplementary Figure 3 [file 41440_2024_1678_MOESM5_ESM.docx]

*Supplementary Figure 3: Pilot study of maternal circulating soluble (pro)renin receptor (s(P)RR) concentrations in pregnant rats following injection of s(P)RR adenovirus.* On day 8 of pregnancy, rats were anaesthetized with 2% isoflurane in oxygen and a single bolus dose (varying dosage) of adenovirus was injected into the tail vein. On day 18 of pregnancy, rats were anaesthetised by 4% isoflurane in oxygen and maternal blood was collected by cardiac puncture. Circulating s(P)RR levels were measured via ELISA. Both the 0.5x10^9 PFU and 1.0x10^9 PFU groups displayed significantly higher s(P)RR levels compared to the 2.0x10^9 PFU group (11914 pg/ml, P<0.001, 9314 pg/ml, P=0.01, respectfully). Due to the consistent increase in s(P)RR concentration seen, a dose of 1.0x10^9 PFU was chosen for the full study. All data are presented as mean +/- SEM. N=1 animal per group performed in technical quadruplicate.
